# CLEC3B (tetranectin) expression is associated with vascular localization and tumor aggressiveness in breast cancer

**DOI:** 10.7150/ijms.132030

**Published:** 2026-07-22

**Authors:** Ching-Ting Wei, Teng-Hung Yu, Chia-Chang Hsu, Chin-Feng Hsuan, Wei-Chin Hung, Chao-Ping Wang, Wei-Hua Tang, Fu-Mei Chung, Yau-Jiunn Lee, Chi-Chang Chang, Chia-Chi Chen

**Affiliations:** 1 Division of General Surgery, Department of Surgery, E-Da Hospital, I-Shou University, Kaohsiung 82445, Taiwan.; 2 The School of Chinese Medicine for Post Baccalaureate, College of Medicine, I-Shou University, Kaohsiung 82445, Taiwan.; 3 Division of Cardiology, Department of Internal Medicine, E-Da Hospital, I-Shou University, Kaohsiung 82445, Taiwan.; 4 School of Medicine, College of Medicine, I-Shou University, Kaohsiung 82445, Taiwan.; 5 Division of Gastroenterology and Hepatology, Department of Internal Medicine, E-Da Hospital, I-Shou University, Kaohsiung 82445, Taiwan.; 6 Health Examination Center, E-Da Dachang Hospital, I-Shou University, Kaohsiung 80794, Taiwan.; 7 Division of Cardiology, Department of Internal Medicine, E-Da Dachang Hospital, I-Shou University, Kaohsiung 807066, Taiwan.; 8 School of Medicine for International Students, College of Medicine, I-Shou University, Kaohsiung 82445, Taiwan.; 9 Division of Cardiology, Department of Internal Medicine, Ministry of Health and Welfare Yuli Hospital, Hualien 98142, Taiwan.; 10 Faculty of Medicine, School of Medicine, National Yang Ming Chiao Tung University, Taipei 112304, Taiwan.; 11 Lee's Endocrinologic Clinic, Pingtung 90000, Taiwan.; 12 Department of Obstetrics & Gynecology, E-Da Hospital, I-Shou University, Kaohsiung 82445, Taiwan.; 13 Department of Obstetrics & Gynecology, E-Da Dachang Hospital, I-Shou University, Kaohsiung 807066, Taiwan.; 14 Department of Pathology, E-Da Hospital, I-Shou University, Kaohsiung 82445, Taiwan.; 15 Department of Physical Therapy, I-Shou University, Kaohsiung 82445, Taiwan.; 16 Department of Occupational therapy, I-Shou University, Kaohsiung 82445, Taiwan.

**Keywords:** Breast cancer, CLEC3B, tetranectin, tumor microenvironment, tumor vasculature

## Abstract

**Background:**

C-type lectin domain family 3 member B (CLEC3B), also known as tetranectin, is a secreted protein associated with extracellular matrix remodeling and tumor microenvironment regulation. However, its expression pattern and clinical significance in breast cancer tissues remain unclear. This study aimed to investigate CLEC3B expression in breast cancer tissues and its associations with clinicopathological characteristics and the tumor microenvironment.

**Methods:**

Ninety-eight women with newly diagnosed breast cancer who underwent surgical treatment at our hospital between September 2023 and December 2024 were included in this study. Tumor and adjacent non-tumorous tissues, including vascular regions, were obtained from the enrolled patients and processed for immunohistochemical analysis. CLEC3B expression was semiquantitatively graded from 1 to 4. Associations between CLEC3B expression, clinicopathological and biochemical variables were analyzed using the chi-square test, Spearman correlation, and multivariable logistic regression. Double immunofluorescence staining was performed to assess colocalization of CLEC3B with CD68 (macrophages) and CD31 (endothelial cells).

**Results:**

The expression of CLEC3B was identified in both tumor cell membrane and cytoplasm, and was significantly higher in breast cancer tissues than in adjacent non-tumorous tissues. CLEC3B expression was also significantly higher in blood vessels than in tumor parenchyma (p < 0.0001), indicating predominant vascular localization. High CLEC3B expression (grade 3-4) was associated with a tumor size of 2-5 cm, Ki-67 ≥14%, and the luminal B HER2-positive subtype. Spearman analysis showed that a high CLEC3B expression was correlated with larger tumor size, higher Ki-67 index, higher carcinoembryonic antigen level, and shorter prothrombin time. Multivariable logistic regression analysis further demonstrated that tumor size ≥2 cm and high Ki-67 (≥14%) were independently associated with high CLEC3B expression, whereas molecular subtype was not. CLEC3B was colocalized with CD68 and CD31, confirming its expression in macrophages and endothelial cells. CD4⁺ and CD8⁺ T-cell infiltration was abundant regardless of CLEC3B expression level.

**Conclusion:**

The expression of CLEC3B was upregulated in breast cancer tissues and predominantly localized to the tumor vasculature and macrophages. Its expression was associated with aggressive clinicopathological features and certain biochemical parameters. No apparent differences in CD4⁺ or CD8⁺ T-cell infiltration were observed across different levels of CLEC3B expression. These findings suggest that CLEC3B expression may be associated with vascular and microenvironment-related characteristics of breast cancer.

## Introduction

Tetranectin, encoded by the C-type lectin domain family 3 member B (CLEC3B) gene, is a plasminogen kringle 4-binding protein localized to plasma, the extracellular matrix, and exosomes, where it participates in extracellular proteolytic processes 1-3. Tetranectin contributes to extracellular proteolysis by promoting plasminogen activation, a process closely linked to tumor invasion and metastasis 1,4,5. Through its role in plasminogen activation, CLEC3B may indirectly contribute to extracellular matrix remodeling, a key process in tumor progression 3-6. In addition to its role in extracellular matrix dynamics, CLEC3B has been implicated in cancer biology and may influence tumor progression by modulating the tumor microenvironment 2,3,7. Previous studies have also suggested that CLEC3B may regulate angiogenesis and metastasis via exosome-associated AMPK and VEGF signaling pathways 2. However, the biological and clinical significance of CLEC3B expression in breast cancer remains poorly understood.

Breast cancer is a heterogeneous disease characterized by distinct molecular subtypes, each associated with different therapeutic responses and clinical outcomes 8,9. Increasing evidence indicates that the tumor microenvironment, including immune components and extracellular matrix remodeling, plays a critical role in tumor progression and metastasis 10,11. Tumor-associated macrophages, stromal fibroblasts, endothelial cells, and proteolytic factors form a complex signaling network that regulates angiogenesis, immune cell infiltration, and tumor invasion 10-13. Given that CLEC3B is involved in plasminogen activation and extracellular matrix interactions, it may serve as a potential mediator linking tissue remodeling, inflammation, and cancer development 1,2,4,5,14. However, few studies have explored its expression pattern in breast cancer tissues or its association with histopathological and immune features.

Accumulating evidence across multiple malignancies indicates that dysregulated CLEC3B expression is associated with tumor progression, metastatic potential, and clinical outcomes 15-17, supporting a context-dependent role for CLEC3B within the tumor microenvironment, potentially involving immune-related regulatory mechanisms 15-17. Consistent with this notion, a previous study reported the downregulation of CLEC3B in triple-negative breast cancer (TNBC), particularly in relapsed tumors, and that higher CLEC3B expression was associated with improved prognosis following chemotherapy. Mechanistically, CLEC3B has been shown to enhance cisplatin (CDDP) sensitivity by promoting ferroptosis, potentially through interactions with metal ion transporters such as SLC39A8 and SLC39A14 18. Given the emerging link between ferroptosis and tumor immunity, these findings may also have implications for immune regulation within the tumor microenvironment. However, the cellular origin of CLEC3B in breast cancer tissues, whether from tumor cells, stromal elements, or infiltrating immune cells, remains unclear. Clarifying its distribution may provide important insights into its biological function and clinical relevance. Therefore, this study aimed to investigate the tissue expression of CLEC3B in patients with primary breast cancer using double immunofluorescence and immunohistochemistry (IHC). We further examined the associations between CLEC3B expression, clinicopathological characteristics, and patterns of immune cell infiltration. Elucidating the localization and clinical significance of CLEC3B may enhance our understanding of its role in the tumor microenvironment and support its potential as a biomarker or therapeutic target in breast cancer.

## Methods

### Study subjects

A total of 98 women (mean age, 53 years; range, 24-86 years) with a new diagnosis of breast cancer who underwent surgical treatment at our hospital from September 2023 to December 2024 were included in this study. The inclusion criteria were: (1) histopathological confirmation of invasive breast cancer; (2) no prior cancer-related treatment, including radiotherapy, chemotherapy, or immunotherapy, before surgery, with patients scheduled to undergo either partial or total mastectomy; and (3) willingness to sign informed consent forms. The exclusion criteria were: (1) a history of mastectomy or previous cancer-related treatment, including chemotherapy, immunotherapy or radiotherapy; and (2) lack of informed consent. The Institutional Review Board of E-Da Hospital approved the study protocol (approval #: EMRP-112-082). Clinical and demographic data were collected from medical records. Tumor staging was assessed according to the American Joint Committee on Cancer (AJCC) staging system.

### Laboratory data

Plasma levels of aspartate aminotransferase (AST) and alanine aminotransferase (ALT) were determined using a Hitachi 7170A parallel multichannel analyzer (Tokyo, Japan), as previously reported 19. The standard clotting method was used to assess prothrombin time. The concentration of carcinoembryonic antigen (CEA) was measured by chemiluminescent microparticle immunoassay. Peripheral total and differential leukocyte (neutrophils, monocytes, and lymphocytes) counts were analyzed with an automated hematology analyzer (XE-2100; Sysmex, Kobe, Japan). Red blood cell-related indices including mean corpuscular hemoglobin concentration, hemoglobin and hematocrit levels were evaluated, along with red cell distribution width (RDW)-standard deviation, RDW-coefficient of variation, and platelet count. Absolute leukocyte subtype counts were calculated by multiplying the total leukocyte count by their respective differential percentages.

### Clinicopathologic characteristics of the tumors

IHC staining for estrogen receptor (ER) and progesterone receptor (PR) was used to confirm the presence of breast cancer. Staging and histology were assessed using the TNM and Bloom-Richardson system, respectively. Subgroup analysis was conducted as follows: age (<50 or ≥50 years), Ki67 level (<14% or ≥14%), AJCC stage (0-II or III-IV), histological grade (1+2 or >3), pathologic T stage (T0+T1+T2 or T3+T4), lymph node metastasis (N0+N1 or N2+N3), tumor size (<2, 2-5, or >5 cm), ER positive or negative, PR positive or negative, and human epidermal growth factor receptor (HER2) positive or negative. Molecular tumor subtypes were determined through IHC analysis of ER, PR, HER2, and Ki-67 20. The subtypes were defined as luminal A, luminal B HER2-negative, and luminal B HER2-positive, HER2-enriched, and triple-negative 20. Luminal A was defined as HER2 negativity, ≥20% ER/PR positivity, and low Ki-67 level (<14%); luminal B HER2-negative was defined as HER2 negativity, ER positivity, and either a negative or low PR expression (<20%) or high Ki-67 expression (≥14%); luminal B HER2-positive was defined as the overexpression or amplification of HER2, ER positivity, and the presence of any level of Ki-67 and PR.

### Tissue sample collection

Tissue samples from cancerous and adjacent noncancerous areas with blood vessels were collected from the enrolled patients, fixed in 10% buffered formalin and embedded in paraffin. Sections with 4-μm thickness were then cut and stained with hematoxylin and eosin for the IHC analysis of PR and ER status. The standard HercepTest procedure (Dako 5204) was used in staining for HER2/neu oncoprotein.

### Analysis of CLEC3B expression

IHC analysis of CLEC3B was performed using a Bond-Max system (Leica Microsystems). Briefly, the prepared tissue samples were placed on slides and left to dry at 60°C for 1 hour. The slides were covered with Bond Universal Covertiles and analysis was conducted using the Bond-Max system (Leica Microsystems) and an Epredia^TM^ TL-125-QHD detection system (Thermo Fisher Scientific Inc.) following the provided instructions: (1) Deparaffinization with three 10-minute changes of xylene; (2) Rehydration with two 5-minute changes of absolute alcohol; (3) Immersion in 95% alcohol for 2 min; (4) Immersion for 2 minutes in 85% alcohol; (5) Immersion for 2 minutes in 75% alcohol; (6) Washing twice in PBS buffer; (7) Blocking endogenous peroxidase activity with hydrogen peroxide block for 10 q; (8) Heat-mediated antigen retrieval for 15 minutes using Tris-EDTA (pH 9.0); (9) Immunoblocking for 5 minutes; (10) Incubation with the CLEC3B primary antibody (Product # MA5-29152, Invitrogen, Thermo Fisher Scientific, Waltham, MA, USA) at a dilution of 1:500 at 37°C for 1 hour, followed by two washes in PBS buffer; (11) Application of the secondary antibody; (12) Preparation of the DAB chromogen solution by mixing 30 μl (1 drop) of DAB Chromogen with 1.0 ml of DAB Buffer, which was then applied for 2 minutes; (13) Counterstaining with hematoxylin for 1 minute, and then washing under running water for 5 minutes; (14) Dehydration through immersion in 95% alcohol and two changes of absolute alcohol, 5 minutes each; (15) Clearing with two changes of xylene, 5 minutes each. Finally, the slides were mounted with a xylene-based mounting medium and observed under a light microscope. In addition, among the 98 patients, we analyzed 59 pairs of breast cancer and non-tumor tissue samples using IHC staining to measure CLEC3B expression levels. To compare the expression of CLEC3B between tissue parenchyma and vessels, staining was evaluated separately in predefined regions of interest with reference to the corresponding hematoxylin and eosin-stained sections. Tumor tissue regions were defined as invasive tumor parenchymal areas, excluding obvious vascular structures, vascular walls, necrotic areas, and large stromal regions. Non-tumorous tissue regions were defined as adjacent non-tumorous breast tissue parenchymal areas, excluding vascular structures. Vascular regions were defined as morphologically identifiable blood vessels, confirmed by comparison with the corresponding hematoxylin and eosin-stained sections, and characterized by a visible vascular lumen and/or endothelial lining with surrounding vascular wall structures. CLEC3B grades were assigned separately for tissue parenchymal regions and vascular regions using the same four-tier semi-quantitative scoring system based on the percentage of positively stained cells or structures.

### IHC staining

The CLEC3B IHC staining results were semi-quantitatively evaluated based on the percentage of positively stained cells. A cell was considered CLEC3B-positive when distinct membranous and/or cytoplasmic DAB staining above background intensity was observed. Weak but definite staining was counted as positive, whereas nonspecific background staining, hematoxylin-only nuclei, necrotic areas, and poorly preserved tissue areas were excluded from scoring. For tumor tissue scoring, the percentage of CLEC3B-positive invasive tumor cells was calculated. For non-tumorous tissue scoring, CLEC3B-positive cells within the selected adjacent non-tumorous areas were evaluated. Vascular staining was evaluated separately and was not included in the tumor parenchymal IHC score. The percentage of CLEC3B-positive cells was then graded on a 4-point scale as follows: grade 1, <25%; grade 2, 25-50%; grade 3, 51-75%; and grade 4, >75% positive cells. All slides were independently assessed by two blinded investigators under identical experimental conditions. Interobserver agreement was evaluated using Cohen's kappa coefficient (κ = 0.831), indicating excellent agreement. Discrepancies were resolved through joint review and consensus. For subsequent analysis, scores were dichotomized into low expression (grades 1-2) and high expression (grades 3-4), based on the median value and consistent with prior studies 21,22. The cutoff was also biologically relevant based on observed differences in clinical outcomes.

### Double immunofluorescence staining

Double immunofluorescence staining was conducted using sections of the paraffin-embedded breast cancer tissue samples. After deparaffinization and rehydration following the standard bright-field IHC protocol, antigen retrieval was carried out in EDTA buffer for 30 minutes. Subsequent blocking steps were performed as in the IHC procedure, followed by incubation of the sections with a mixture of primary antibodies in a humidified chamber at 37°C for 1 hour: CLEC3B (MA5-29152, 1:100) together with either CD31 (GTX20218, 1:100) or CD68 (GTX41868, 1:100). After washing with PBS, the sections were incubated with corresponding fluorescent secondary antibodies at room temperature for 30 minutes: anti-rabbit Rb-iFluor 594 (Leadgene 24001, 1:500) for CLEC3B, and anti-mouse Ms-iFluor 488 (Invitrogen A11029, 1:500) for CD31 or CD68. The sections were then washed and mounted using a fluorescent mounting medium containing DAPI (or according to the laboratory's standard IHC mounting procedure). All other steps, including blocking, washing, and mounting, followed the same conditions as bright-field IHC. To confirm antibody specificity, isotype or single-staining controls were included. Fluorescent images were acquired using a confocal microscope in sequential scanning mode to avoid channel crosstalk.

### Statistical analysis

Categorical variables are presented as frequencies and percentages, and differences between groups were analyzed using the chi-square test or Fisher's exact test, as appropriate. Associations between CLEC3B expression and selected clinical or biochemical variables were examined using Spearman's rank correlation analysis, when appropriate. Molecular tumor subtype was not included in the Spearman correlation analysis because it is a categorical variable without an inherent ordinal scale. To identify factors associated with high CLEC3B expression (IHC score 3-4 vs. 1-2), univariate logistic regression analysis was first performed. Variables with clinical relevance and/or statistical significance in the univariate analysis were subsequently included in a multivariable logistic regression model. Given the number of events (n = 37), the number of covariates included in the multivariable model was limited according to the events-per-variable principle to minimize the risk of overfitting. Odds ratios (ORs) and 95% confidence intervals (CIs) were calculated. The statistical analysis was performed using JMP software, version 10.0 for Windows (SAS Institute Inc., Cary, NC, USA), and a two-tailed p value <0.05 was considered statistically significant.

## Results

### Clinicopathological characteristics of the patients

Of the enrolled patients, 54.1% were ≥50 years old, 18.4% had pathologic stage T3 or T4, 15.3% had N2 or N3 lymph node metastasis, and 66.3% had tumors ≥2 cm in size (Table 1). Regarding the molecular tumor subtype, 33.7% of the patients were luminal A, 27.6% luminal B HER2-negative, 15.3% luminal B HER2-positive, 6.1% HER2-enriched, and 17.3% triple-negative.

### IHC staining of CLEC3B in tumor tissues, non-tumorous tissues, and blood vessels

**Figure 1:**
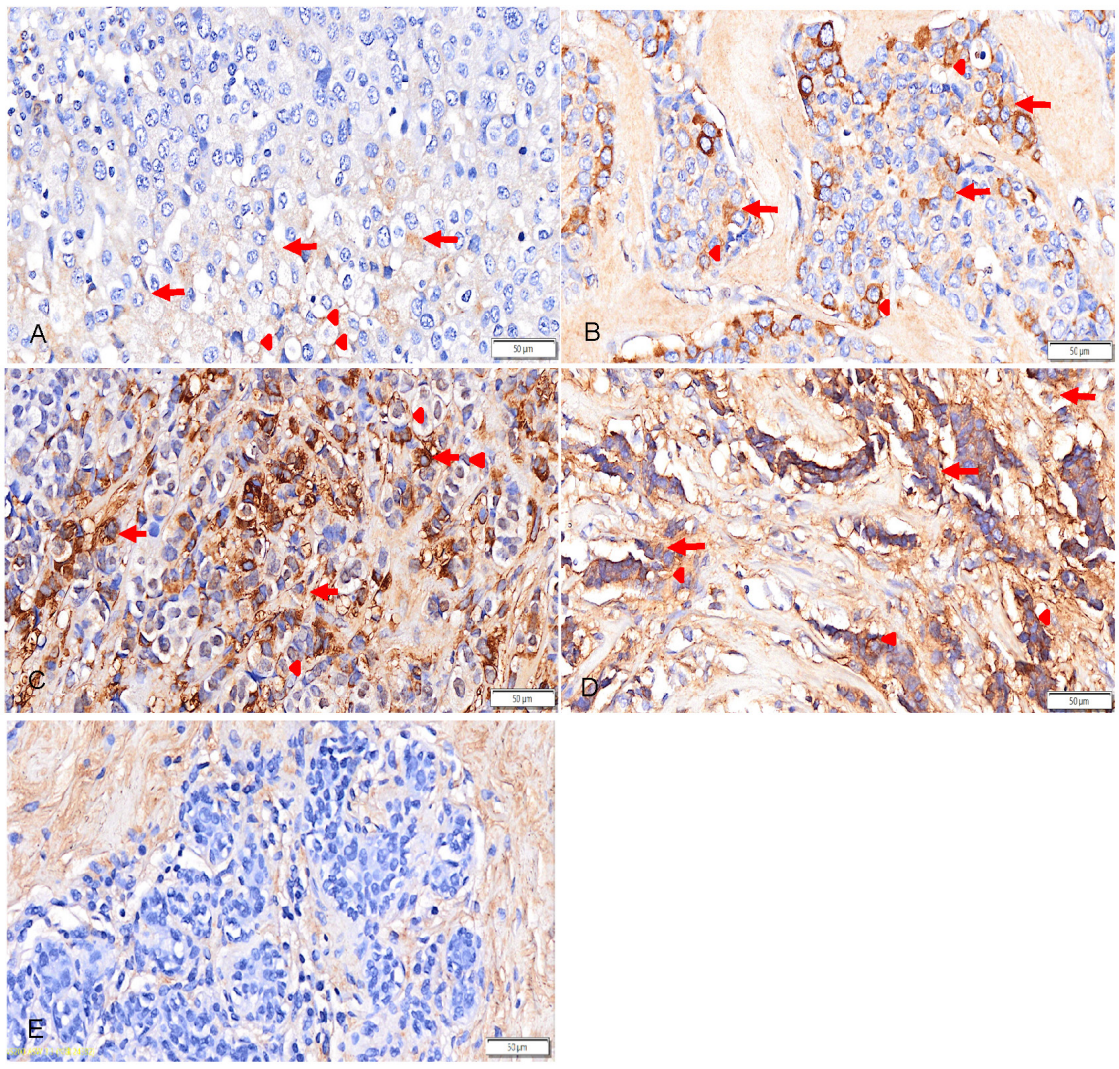
Representative immunohistochemical staining of C-type lectin domain family 3 member B (CLEC3B) in breast cancer tissue specimens and non-tumor tissue. CLEC3B immunoreactivity was evaluated using a four-tier grading system: grade 1 (A), grade 2 (B), grade 3 (C), grade 4 (D), and non-tumor tissue (E). Higher-magnification representative images are shown to better illustrate the membranous and cytoplasmic staining patterns of CLEC3B. Arrowheads indicate membranous staining, and arrows indicate cytoplasmic staining. Scale bars = 50 μm.

**Figure 2:**
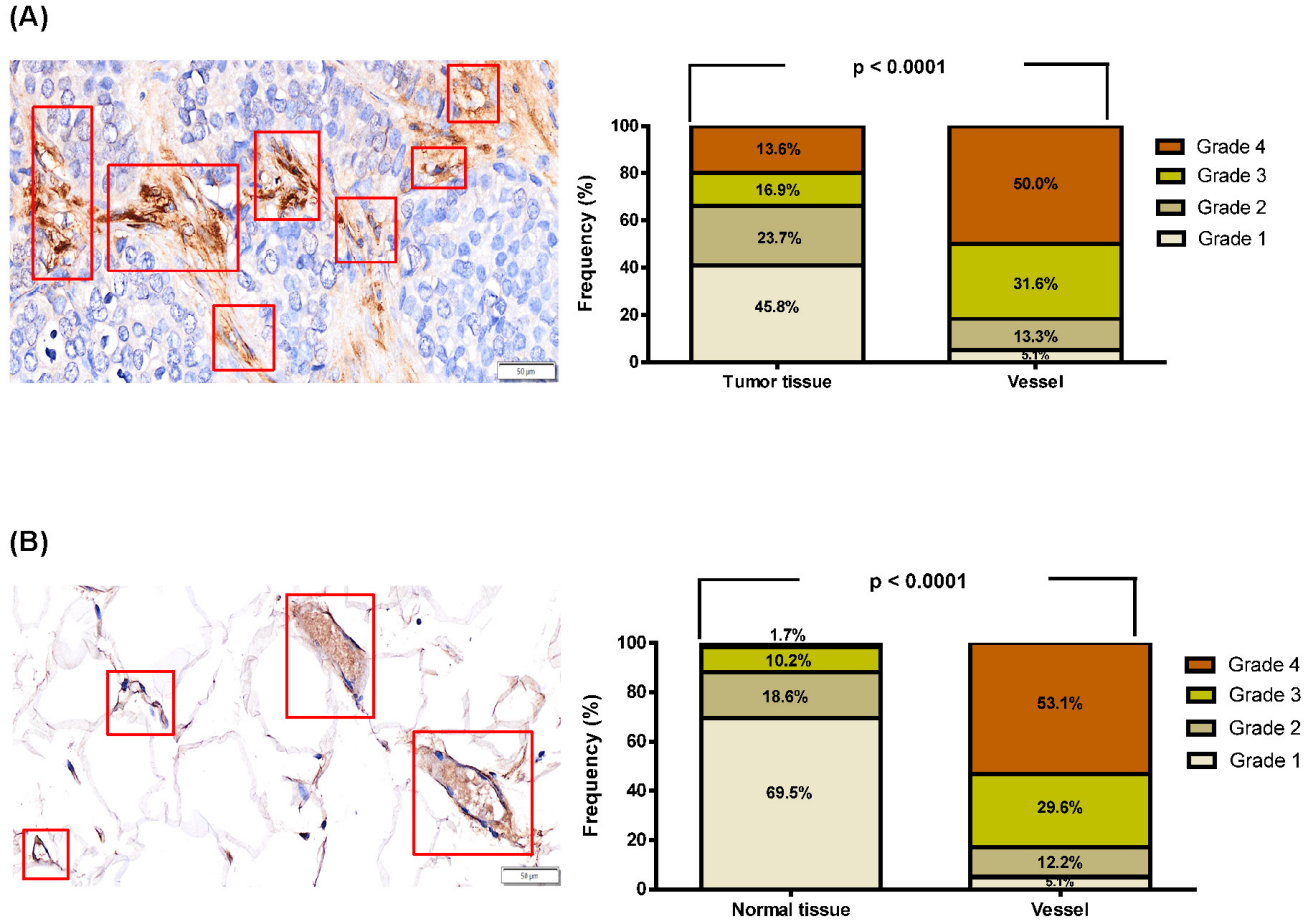
Expression of C-type lectin domain family 3 member B (CLEC3B) in breast cancer tissues, normal tissues, and blood vessels. (A) Representative immunohistochemical staining of CLEC3B in breast cancer tissues and blood vessels. (B) Representative immunohistochemical staining of CLEC3B in normal tissues and blood vessels. Red boxes indicate vascular regions selected for vessel scoring, which were identified based on morphology and reference to the corresponding hematoxylin and eosin-stained sections. CLEC3B staining was evaluated separately in tissue parenchymal regions and vascular regions. CLEC3B was highly expressed in blood vessels regardless of whether they were located in breast cancer or normal tissues. The p-value was calculated using the chi-square test. Scale bar = 50 μm.

CLEC3B immunoreactivity in tumor and non-tumorous tissues was graded using a four-tier scoring system, from grade 1 to grade 4. In the full cohort of 98 breast cancer patients, the distribution of CLEC3B IHC grades in tumor tissues was as follows: grade 1, 36 cases (36.7%); grade 2, 25 cases (25.5%); grade 3, 16 cases (16.3%); and grade 4, 21 cases (21.4%). For subsequent analyses, CLEC3B expression was dichotomized into low expression (grades 1-2, n = 61) and high expression (grades 3-4, n = 37). As shown in Figure 1, CLEC3B expression was observed in both the cell membrane and cytoplasmic regions of breast cancer tissue specimens, with representative membranous and cytoplasmic staining patterns indicated by arrowheads and arrows, respectively. Among the 59 paired tumor and non-tumorous tissue samples, the proportion of samples classified as CLEC3B grade 1 was significantly lower in tumor tissues than in non-tumorous tissues, whereas the proportion classified as grade 4 was significantly higher in tumor tissues (Table 2). Furthermore, CLEC3B expression was significantly higher in blood vessels than in tumor tissues (p < 0.0001, Figure 2A). Similarly, in normal tissues, CLEC3B expression was also significantly higher in blood vessels than in the surrounding normal tissues (p < 0.0001, Figure 2B). These findings indicated that CLEC3B was highly expressed in blood vessels regardless of whether they were located in tumorous or normal tissues, suggesting that CLEC3B may play a specific role in the vascular microenvironment.

### CLEC3B expression and clinicopathological characteristics

We then analyzed the patients' clinicopathological characteristics according to CLEC3B expression in tumor tissues. Compared to the patients with CLEC3B grade 1-2 expression, those with CLEC3B grade 3-4 expression had a higher prevalence of tumor size between 2 and 5 cm, Ki-67 ≥14%, HER2-positive status, and the luminal B HER2-positive molecular subtype. In contrast, smaller tumor size (<2 cm), Ki-67 <14%, HER2-negative status, and the luminal A molecular subtype were more frequently observed in patients with CLEC3B grade 1-2 expression. However, no significant differences were found between the two groups in age, histological grade, pathological T stage, lymph node metastasis, AJCC stage, ER status, or PR status (Table 3).

### Associations between biochemical and clinical variables and CLEC3B expression

Spearman correlation analysis was performed to evaluate the associations between clinicopathological and biochemical variables and CLEC3B IHC score. A higher CLEC3B IHC score was positively correlated with tumor size ≥2 cm (r = 0.252, p = 0.020), Ki-67 ≥14% (r = 0.239, p = 0.031), and CEA level (r = 0.234, p = 0.032). In addition, CLEC3B expression was negatively correlated with prothrombin time (r = -0.224, p = 0.048). No significant associations were found between CLEC3B IHC score and pathologic T stage, AJCC stage, histologic grade, liver function parameters (AST, ALT, APRI), or hematological indices, including white blood cell count, differential leukocyte counts, red blood cell indices, platelet count, and RDW (all p > 0.05) (Table 4).

### Multivariable analysis

To further determine whether these associations were independent, multivariable logistic regression analysis was performed. The analysis demonstrated that tumor size ≥2 cm (OR 4.06, 95% CI 1.44-12.82, p = 0.008) and high Ki-67 (≥14%) (OR 5.35, 95% CI 1.38-26.50, p = 0.015) were independently associated with high CLEC3B expression. In contrast, molecular subtype was not significantly associated with CLEC3B expression (p = 0.861) (Table 5).

### CLEC3B was expressed by macrophages and endothelial cells in breast cancer tissue

**Figure 3:**
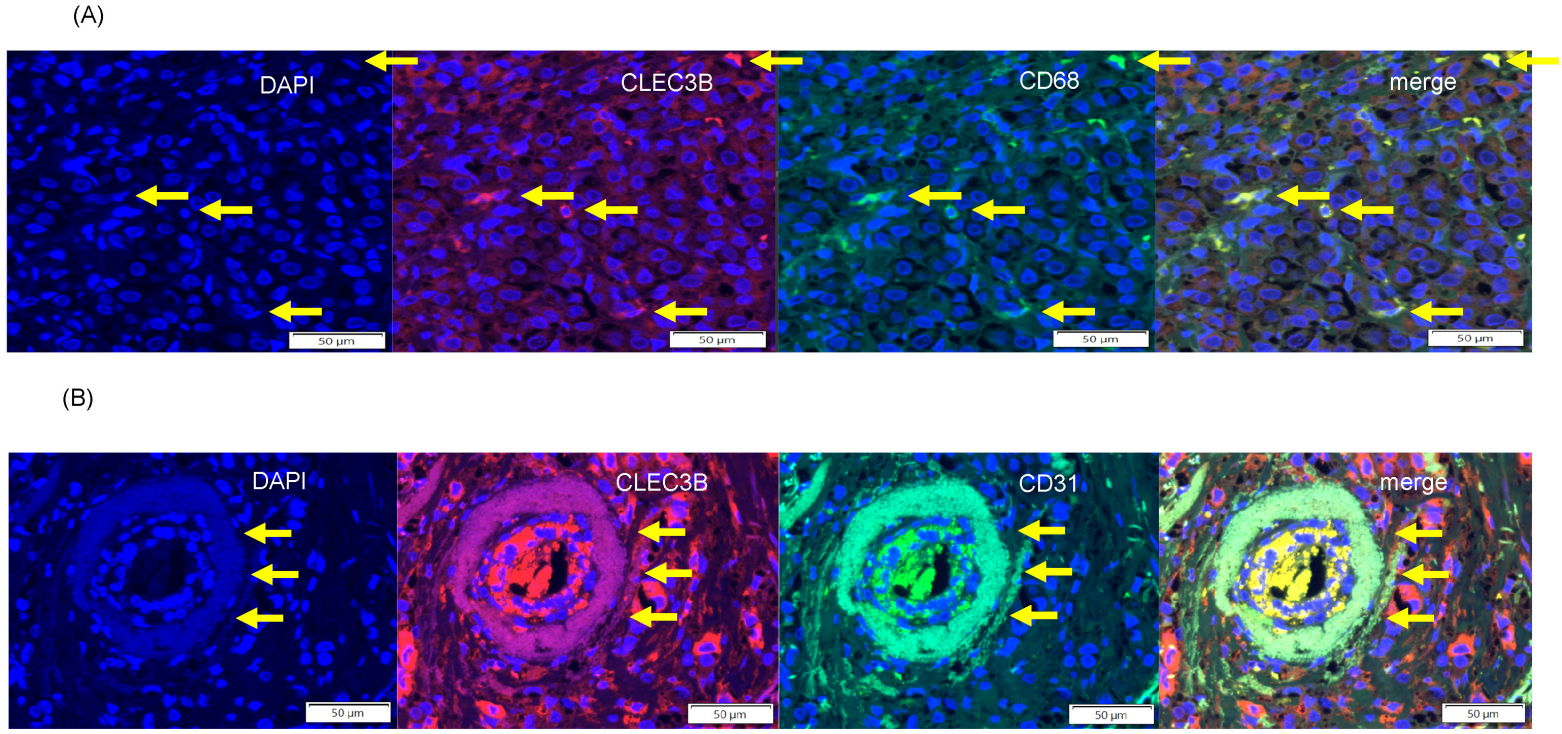
Double immunofluorescence demonstrated colocalization of C-type lectin domain family 3 member B (CLEC3B) with the macrophage marker cluster of differentiation 68 (CD68) (upper panel) and the pan-endothelial cell marker CD31 (lower panel). Nuclei were stained with 4′,6-diamidino-2-phenylindole (DAPI). Scale bars = 50 μm.

Double immunofluorescence showed complete colocalization of the macrophage marker CD68 with CLEC3B, supporting that macrophages express CLEC3B (Figure 3A). In addition, colocalization of CLEC3B with the pan-endothelial marker CD31 supported that endothelial cells also express CLEC3B (Figure 3B).

### Relationships between CLEC3B expression and CD4+ and CD8+ immune cells in breast cancer tissue

**Figure 4:**
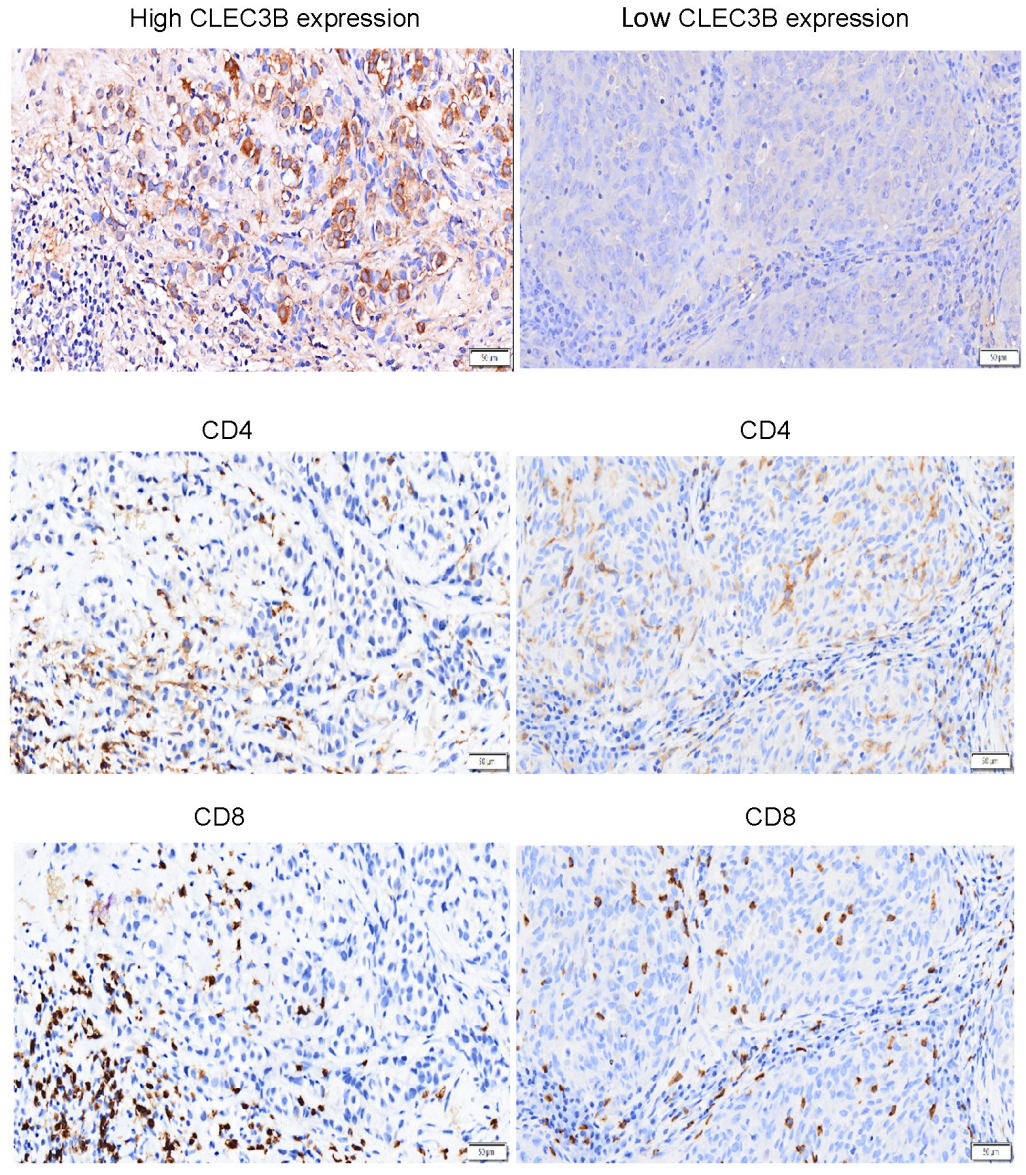
Representative immunohistochemical staining showing CLEC3B protein expression and CD4+ and CD8+ cells in breast cancer samples. CD4+ and CD8+ cells were highly expressed in breast cancer tissues with both a high and low CLEC3B expression.

We then explored relationships between CLEC3B expression and CD4+ and CD8+ immune cells in the breast cancer tissue specimens. The results showed that CD4+ and CD8+ cells were highly expressed in breast cancer tissues with both high and low CLEC3B expressions (Figure 4). These findings illustrate the complexity of the immune microenvironment in breast cancer, and suggest that CD4+ and CD8+ cells may simultaneously participate in processes such as immune surveillance or immune evasion, while the expression of CLEC3B does not directly affect their quantity or distribution.

## Discussion

In this study, we analyzed the expression of CLEC3B in breast cancer tissues and associations with clinicopathological characteristics and composition of the tumor immune microenvironment. There were five major findings. (1) IHC showed that CLEC3B was localized to both the cell membrane and cytoplasm of tumor cells, and that its expression was significantly higher in tumorous tissues compared to adjacent non-tumorous tissues. Its expression was also significantly higher in blood vessels than in the tumor parenchyma (p<0.0001), indicating predominant vascular localization. (2) High IHC scores (grade 3-4) were more frequently observed in the patients with a tumor size of 2-5 cm, Ki-67 ≥14%, and the luminal B HER2-positive subtype, whereas lower scores (grade 1-2) were associated with a tumor size <2 cm and the luminal A HER2-negative subtype. (3) Spearman correlation analysis demonstrated that an elevated CLEC3B expression was significantly associated with a larger tumor size, higher Ki-67 index, increased CEA level, and shorter prothrombin time. Importantly, multivariable analysis further showed that tumor size (≥2 cm) and high Ki-67 (≥14%) remained independently associated with CLEC3B expression, whereas molecular subtype was not statistically significantly associated with CLEC3B expression. The relatively wide confidence intervals observed in the multivariable analysis likely reflect the limited sample size; therefore, these findings should be interpreted with caution. Further studies with larger cohorts are warranted to validate these results. (4) Double immunofluorescence demonstrated that CLEC3B was colocalized with CD68 and CD31, confirming its expression in macrophages and endothelial cells. (5) Both CD4⁺ and CD8⁺ T cells were abundant in tissues with either a high or low CLEC3B expression, suggesting that CLEC3B level does not directly influence T-cell infiltration. These findings provide new insights into the potential role of CLEC3B in the vascular and immune landscapes of breast cancer.

The first key finding is that CLEC3B protein was detected in both the membrane and cytoplasm of tumor cells (Figure 1), with a significantly higher expression in breast cancer tissues compared with adjacent non-tumorous tissues (Table 2). A previous study reported tetranectin expression in normal breast tissue, with strong staining particularly observed in connective tissue fibers 14. Consistent with these findings, CLEC3B immunoreactivity was detectable in non-tumor breast tissues in the present study. In our cohort, 41 of 59 non-tumor samples were classified as grade 1, 11 as grade 2, 6 as grade 3, and 1 as grade 4, indicating variable but present CLEC3B expression in non-tumorous tissues. The representative non-tumorous image shown in Figure 1E illustrates CLEC3B positivity in stromal/connective tissue components and is consistent with the connective tissue-associated staining pattern described previously. This variability in staining intensity may reflect differences in stromal composition, tissue sampling, and the distribution of connective tissue fibers in the examined regions. Importantly, although CLEC3B expression was detectable in non-tumorous stromal/connective tissue components, high-grade CLEC3B expression was more frequently observed in tumor tissues than in non-tumorous tissues in our semi-quantitative analysis. These findings suggest that CLEC3B may be involved in tumor-related biological processes rather than being an incidental finding. Its subcellular distribution is consistent with a potential role in extracellular matrix interactions and cell-microenvironment signaling, both of which are essential for tumor progression and intercellular communication in the tumor microenvironment 10,11. Notably, CLEC3B expression was more prominent in vascular structures than in the tumor cell compartment, indicating preferential vascular localization. This distribution suggests a close association with the tumor vasculature and raises the possibility that CLEC3B may participate in angiogenesis or vascular remodeling. Given that the vasculature serves as a key interface for immune cell trafficking, this distribution suggests a potential role for CLEC3B in mediating interactions between tumor and immune system 10,23. In addition, CLEC3B expression in endothelial compartments may be relevant to vascular-immune crosstalk in the context of an immunosuppressive microenvironment 24,25. Interestingly, vascular enrichment of CLEC3B was observed in both tumor and non-tumorous tissues (Figure 2), suggesting that its expression may be associated with intrinsic vascular biology rather than being tumor-specific. This is supported by previous studies showing that CLEC3B is involved in extracellular matrix remodeling, plasminogen activation, and vascular homeostasis 2,3,26. However, its upregulation in tumor tissues indicates that it may be differentially regulated under malignant conditions, potentially in response to hypoxia, inflammation, or stromal signaling, all of which are well-recognized drivers of gene expression within the tumor microenvironment 10,23,27. Taken together, these findings support a context-dependent role of CLEC3B within the tumor microenvironment, particularly at the interface between tumor cells and the vascular compartment. Nevertheless, its functional significance in tumor angiogenesis and immune regulation remains to be elucidated, and further mechanistic studies are required to determine whether its vascular localization directly influences tumor progression or immune cell dynamics 28,29.

The second key finding of this study is that high tumor CLEC3B expression was associated with more aggressive tumor features, including larger tumor size and increased proliferative activity as reflected by a higher Ki-67 index. These associations remained significant in multivariable analysis, suggesting that CLEC3B expression is independently associated with tumor size and cellular proliferation. In categorical comparisons, high CLEC3B expression was more frequently observed in HER2-positive tumors and in the luminal B HER2-positive subtype; however, molecular subtype was not included in the Spearman correlation analysis because it is a categorical variable without an inherent ordinal scale. In the multivariable analysis, molecular subtype was not retained as an independent determinant of CLEC3B expression. Instead, CLEC3B expression appeared to be more closely related to proliferative and growth-related tumor characteristics rather than intrinsic subtype classification. This observation is consistent with prior studies showing that Ki-67 is a robust marker of tumor proliferation and is strongly associated with tumor aggressiveness in breast cancer 30-32. Given the observational nature of this study, these findings should be interpreted as associations rather than causal relationships. Nevertheless, the consistent association between CLEC3B expression and markers of tumor aggressiveness raises the possibility that CLEC3B may be involved in tumor progression or reflect underlying biological processes associated with rapid tumor growth. Further mechanistic studies are warranted to elucidate the biological role of CLEC3B in breast cancer.

Interestingly, our results demonstrated that CLEC3B expression was significantly higher in breast cancer tissues compared with adjacent non-tumorous tissues, which is inconsistent with previous studies showing the downregulation of CLEC3B in malignancies including lung cancer and triple-negative breast cancer 15,16,18. Several factors may explain this discrepancy. First, CLEC3B expression may be tumor type-specific and context-dependent. While prior studies have reported the downregulation of CLEC3B in multiple cancers, its expression pattern may vary according to tumor biology, molecular subtype, and the tumor microenvironment. Notably, previous studies have focused primarily on triple-negative breast cancer, whereas our cohort included a broader spectrum of subtypes with distinct biological characteristics 8,33. Second, methodological differences may contribute to the inconsistent findings. Most prior studies have relied on transcriptomic data, whereas our study assessed protein expression using IHC, enabling evaluation of spatial localization. Discrepancies between mRNA and protein expression levels are well recognized and may reflect post-transcriptional regulation and protein stability 37. In our study, CLEC3B was enriched in vascular structures and stromal components, suggesting that its expression extends beyond tumor cells and may contribute to the overall increased protein levels observed in tumor tissues. Third, CLEC3B may play different roles in different cellular compartments within the tumor microenvironment. We observed the expression of CLEC3B in endothelial cells and macrophages, indicating that it may be associated with microenvironmental remodeling rather than tumor cell-intrinsic expression alone. This is consistent with previous studies highlighting the role of stromal and immune components in shaping tumor behavior and biomarker expression 10,11. Taken together, these findings suggest that CLEC3B expression is highly context-dependent, with potentially distinct roles across tumor types and cellular compartments. Further studies integrating transcriptomic, proteomic, and functional approaches are required to clarify its biological role in breast cancer.

The third key finding of this study is that higher CLEC3B expression was associated with higher CEA level and shorter prothrombin time. The positive association between CLEC3B expression and CEA, a well-established tumor marker, may suggest a link between CLEC3B and tumor-related biological activity. CEA is widely used as a surrogate marker of tumor progression and has been associated with tumor aggressiveness and poor prognosis in several malignancies 35,36. Accordingly, this relationship raises the possibility that CLEC3B may be involved in tumor-associated pathways or reflect alterations in the tumor microenvironment. In addition, the inverse relationship between CLEC3B expression and prothrombin time suggests a potential association with coagulation status. A shorter prothrombin time may indicate a relatively procoagulant state, which is frequently observed in cancer and has been implicated in tumor growth, angiogenesis, and metastasis 37,38. Tetranectin has been reported to participate in plasminogen activation, fibrinolysis, and extracellular matrix remodeling 3, providing a potential mechanistic link between CLEC3B expression and coagulation-related processes observed in this study. Taken together, these findings suggest that CLEC3B may function at the intersection of tumor biology and coagulation pathways. However, the underlying mechanisms remain unclear, and further studies are needed to determine whether CLEC3B directly modulates coagulation processes or serves as a biomarker of cancer-associated systemic alterations.

The fourth key finding of this study is the colocalization of CLEC3B with CD68 and CD31, suggesting potential localization of CLEC3B within macrophages and endothelial cells, respectively. CD68 is a well-established marker of macrophages 39, while CD31 is commonly used to identify endothelial cells and vascular structures 40. Our findings suggest that CLEC3B may be distributed across both immune and vascular compartments within the tumor microenvironment. Macrophages are key regulators of inflammation, tumor progression, and tissue remodeling, while endothelial cells play central roles in angiogenesis, vascular homeostasis, and immune regulation 41-43. The presence of CLEC3B in these cell types raises the possibility that it may be associated with processes related to immune regulation and vascular function. Its localization in macrophages and endothelial cells suggests that it may be linked to microenvironmental processes such as matrix remodeling, cell migration, and angiogenesis. Furthermore, interactions between macrophages and endothelial cells are critical for tumor progression and metastasis through the coordinated regulation of inflammation and angiogenesis 44-46. Therefore, the observed colocalization of CLEC3B with CD68 and CD31 may reflect its involvement in macrophage-endothelial crosstalk within the tumor microenvironment. However, the functional role of CLEC3B in these cell populations remains unclear, and further studies are required to determine whether it directly modulates macrophage activation, endothelial function, or macrophage-endothelial interactions.

The fifth important finding of this study is that CD4⁺ and CD8⁺ T-cell infiltration appeared abundant across tumors regardless of CLEC3B expression levels, with no clear differences observed between groups. However, it should be noted that T-cell infiltration was evaluated qualitatively based on IHC observations rather than quantitative analysis, and subtle differences in T-cell density may therefore not have been fully captured. Although CLEC3B has been implicated in extracellular matrix remodeling and plasminogen activation, both of which can influence immune cell trafficking, our findings suggest that these mechanisms may not be the primary determinants of T-cell infiltration in this context. Instead, T-cell recruitment is more likely regulated by chemokine signaling pathways, such as the CXCL9/CXCL10-CXCR3 axis, and antigen presentation dynamics 42,47,48. The presence of abundant tumor-infiltrating lymphocytes irrespective of CLEC3B expression further suggests that CLEC3B protein expression may have a limited association with T-cell density, while potentially contributing to other aspects of the tumor microenvironment, such as angiogenesis, stromal remodeling, or innate immune regulation. Alternatively, CLEC3B may influence qualitative features of T-cell function, including activation status, exhaustion, or cytokine production, rather than their absolute abundance. Taken together, these findings highlight that CLEC3B is unlikely to be a major determinant of T-cell infiltration density, but may still be relevant to functional immune modulation. Further studies are warranted to clarify its role in shaping the functional phenotypes of tumor-infiltrating lymphocytes.

This study has several important limitations. First, it is a single-center retrospective study with a limited sample size. Although significant associations between CLEC3B expression and clinicopathological features were identified, potential selection bias cannot be excluded, and the generalizability of the findings may be limited. Validation in larger, multicenter cohorts is warranted. Second, CLEC3B expression was assessed using IHC with semi-quantitative scoring based on the percentage of CLEC3B-positive cells. This percentage-based scoring system was chosen to provide a reproducible and clinically interpretable assessment of CLEC3B expression across tumor and non-tumorous tissue compartments, and all slides were independently evaluated by two blinded investigators with excellent interobserver agreement. However, this scoring method did not incorporate staining intensity, positive staining area, or integrated optical density. Because CLEC3B protein is secreted and may accumulate in extracellular, stromal, and vascular compartments, staining intensity and positive area may better reflect its relative abundance within the tissue microenvironment. Moreover, CLEC3B immunostaining was performed using a single primary antibody targeting CLEC3B protein. Although detailed antibody information and positive and negative controls were provided to support staining specificity, validation with an independent antibody targeting a different epitope was not performed because of the limited availability of remaining tissue sections. Future studies using H-score, integrated optical density, digital image analysis, independent antibodies, or orthogonal validation methods are needed to provide a more quantitative assessment and to confirm the specificity and reproducibility of CLEC3B staining. Third, this study was observational in nature and therefore cannot establish causal relationships. Although CLEC3B expression was associated with multiple clinicopathological features, it remains unclear whether CLEC3B directly contributes to these biological processes or reflects secondary changes within the tumor microenvironment. Functional studies are required to clarify its biological role in breast cancer. Fourth, although CLEC3B expression was detected in macrophages and endothelial cells, the functional states of these cell populations were not characterized. In addition, T-cell infiltration was assessed qualitatively rather than quantitatively, which may have limited the ability to detect subtle differences in immune cell density. Given the heterogeneity of tumor-associated macrophages and endothelial cells, further studies are needed to determine whether CLEC3B expression is associated with specific cellular phenotypes or functional programs. Finally, due to the lack of longitudinal follow-up data, survival and prognostic analyses could not be performed. Therefore, although CLEC3B may have potential as a biomarker, its prognostic and predictive significance remains unclear. Future studies incorporating long-term follow-up and clinical outcomes are needed to better define its clinical relevance in breast cancer.

## Conclusion

The results of this study showed that CLEC3B was upregulated in breast cancer tissues and predominantly localized in vascular structures, with additional expression observed in tumor cells and macrophages. Elevated CLEC3B expression was associated with larger tumor size and increased proliferative activity, supporting a potential link with tumor aggressiveness. Colocalization with CD68 and CD31 further suggests localization of CLEC3B within immune and vascular compartments of the tumor microenvironment. Despite its reported involvement in extracellular matrix remodeling, no clear differences in CD4⁺ or CD8⁺ T-cell infiltration were observed across different levels of CLEC3B expression, indicating that it may not be a primary determinant of adaptive immune cell recruitment. Instead, CLEC3B expression may be more closely related to vascular features, stromal remodeling, or innate immune components. Overall, these findings suggest that CLEC3B may contribute to interactions between tumor cells and stromal components in breast cancer. Further studies are needed to elucidate its biological role and to determine whether it may serve as a clinically relevant biomarker or potential therapeutic target.

## Figures and Tables

**Table 1:** Clinicopathological characteristics of the patients with breast cancer (n = 98)

Variable	Category	n (%)
Age (years)	<50	45 (45.9)
	≥50	53 (54.1)
Tumor size (cm)	<2	33 (33.7)
	2-5	48 (49.0)
	>5	17 (17.3)
Pathologic T stage	T0+T1+T2	80 (81.6)
	T3+T4	18 (18.4)
Lymph node metastasis	N0+N1	83 (84.7)
	N2+N3	15 (15.3)
Histologic grade	1+2	52 (53.1)
	>3	46 (46.9)
AJCC Stage	0-II	73 (74.5)
	III-IV	25 (25.5)
Ki67 status	<14%	39 (39.8)
	≥14%	59 (60.2)
Estrogen receptor	Negative	18 (18.4)
	Positive	80 (81.6)
Progesterone receptor	Negative	33 (33.7)
	Positive	65 (66.3)
HER2	Negative	75 (76.5)
	Positive	23 (23.5)
Molecular tumor subtype	1 (Luminal A)	33 (33.7)
	2 (Luminal B HER2-negative)	27 (27.6)
	3 (Luminal B HER2-positive)	15 (15.3)
	4 (HER2-enriched)	6 (6.1)
	5 (Triple-negative)	17 (17.3)

AJCC, American Joint Committee on Cancer.

**Table 2:** Immunohistochemical staining for C-type lectin domain family 3 member B (CLEC3B) in tumor and non-tumorous tissues.

IHC score	Tumor	Non-tumor	p-value
No	59	59	
Grade 1	27(45.8)	41(69.5)	0.015
Grade 2	14(23.7)	11(18.6)	0.653
Grade 3	10(16.9)	6(10.2)	0.421
Grade 4	8(13.6)	1(1.7)	0.032

The C-type lectin domain family 3 member B (CLEC3B) IHC staining was scored from 1 to 4 based on the percentage of positively stained cells as follows: grade 1 for <25%, grade 2 for 25-50%, grade 3 for 51-75%, and grade 4 for >75% positive cells. IHC, immunohistochemistry.

**Table 3:** Expression of C-type lectin domain family 3 member B (CLEC3B) grouped according to categorical variables.

Parameter	CLEC3B grade 1, 2 N (%)	CLEC3B grade 3, 4 N (%)	p-value
Number	61	37	
Age (years)			
<50	32(52.5)	13(35.1)	0.125
≥50	29(47.5)	24(64.9)	
Tumor size (cm)			
<2	26(42.6)	7(18.9)	**0.033**
2-5	24(39.3)	24(64.9)	**0.026**
>5	11(18.0)	6(16.2)	0.870
Pathologic T stage			
T0+T1+T2	49(80.3)	31(83.8)	0.802
T3+T4	12(19.7)	6(16.2)	
Lymph node metastasis			
N0+N1	55(90.2)	28(75.7)	0.084
N2+N3	6(9.8)	9(24.3)	
Histologic grade			
1+2	35(57.4)	17(45.9)	0.304
>3	26(42.6)	20(54.1)	
American Joint Committee on Cancer stage			
0-II	48(78.7)	25(67.6)	0.232
III-IV	13(21.3)	12(32.4)	
Ki67 status			
<14%	30(49.2)	9(24.3)	**0.029**
≥14%	31(50.8)	28(75.7)	
Estrogen receptor			
Negative	11(18.0)	7(18.9)	0.889
Positive	50(82.0)	30(81.1)	
Progesterone receptor			
Negative	18(29.5)	15(40.5)	0.326
Positive	43(70.5)	22(59.5)	
HER2			
Negative	51(83.6)	24(64.9)	**0.048**
Positive	10(16.4)	13(35.1)	
Molecular tumor subtype			
Luminal A	26(42.6)	7(18.9)	**0.033**
Luminal B HER2-negative	15(24.6)	12(32.4)	0.615
Luminal B HER2-positive	6(9.8)	9(24.3)	**0.048**
HER2-enriched	3(4.9)	3(8.1)	0.666
Triple-negative	11(18.0)	6(16.2)	0.738

**Table 4:** Spearman correlation analysis of clinical and biochemical variables with the expression and concentration of C-type lectin domain family 3 member B (CLEC3B).

	CLEC3B IHC score 3, 4	
Parameter	r	p-value
Tumor size (≥2 cm versus <2 cm)	0.252	**0.020**
Pathologic T stage (T3+T4 versus T0+T1+T2)	-0.028	0.805
AJCC stage (III-IV versus 0-II)	0.138	0.238
Ki67 (≥14% versus <14%)	0.239	**0.031**
Histologic grade (>3 versus1+2)	0.121	0.274
AST	-0.003	0.976
ALT	0.005	0.965
APRI	-0.062	0.590
CEA	0.234	**0.032**
Prothrombin time	-0.224	**0.048**
White blood cell count	-0.097	0.385
Neutrophil count	-0.121	0.380
Monocyte count	-0.157	0.252
Lymphocyte count	-0.045	0.747
Red blood cells	0.002	0.983
Hemoglobin	-0.057	0.609
Hematocrit	-0.030	0.786
MCH	-0.077	0.488
MCHC	-0.100	0.368
Platelet count	0.127	0.254
RDW-SD	0.126	0.255
RDW-CV	-0.024	0.828

C-type lectin domain family 3 member B (CLEC3B); AJCC, American Joint Committee on Cancer; AST, Aspartate transaminase; ALT, Alanine transaminase; APRI, AST to platelet ratio index; CEA, carcinoembryonic Antigen; MCH, mean corpuscular hemoglobin; MCHC, mean corpuscular-hemoglobin concentration; RDW, red cell distribution width; SD, standard deviation; CV, coefficient of variation.

**Table 5:** Multivariable logistic regression analysis of factors associated with high CLEC3B expression (IHC 3-4 vs. 1-2).

Variables	OR (95% CI)	p-value
Tumor size (≥2 cm versus <2 cm)	4.06 (1.44-12.82)	0.008
Ki67 (≥14% versus <14%)	5.35 (1.38-26.50)	0.015
Molecular subtype (non-luminal A vs. luminal A)	1.15 (0.21-5.25)	0.861

Variables included in the model were selected based on clinical relevance and univariate analysis results, with the number of covariates limited according to the events-per-variable principle to avoid overfitting. OR, odds ratio; CI, confidence interval.
